# Robust and Fast Scene Recognition in Robotics Through the Automatic Identification of Meaningful Images

**DOI:** 10.3390/s19184024

**Published:** 2019-09-18

**Authors:** David Santos, Eric Lopez-Lopez, Xosé M. Pardo, Roberto Iglesias, Senén Barro, Xosé R. Fdez-Vidal

**Affiliations:** CiTIUS Research Centre, University of Santiago de Compostela, 15782 Santiago de Compostela, Spain; eric.lopez@udc.es (E.L.-L.); xose.pardo@usc.es (X.M.P.); senen.barro@usc.es (S.B.); xose.vidal@usc.es (X.R.F.-V.)

**Keywords:** scene recognition, image collection summarization, meaningful images

## Abstract

Scene recognition is still a very important topic in many fields, and that is definitely the case in robotics. Nevertheless, this task is view-dependent, which implies the existence of preferable directions when recognizing a particular scene. Both in human and computer vision-based classification, this actually often turns out to be biased. In our case, instead of trying to improve the generalization capability for different view directions, we have opted for the development of a system capable of filtering out noisy or meaningless images while, on the contrary, retaining those views from which is likely feasible that the correct identification of the scene can be made. Our proposal works with a heuristic metric based on the detection of key points in 3D meshes (Harris 3D). This metric is later used to build a model that combines a Minimum Spanning Tree and a Support Vector Machine (SVM). We have performed an extensive number of experiments through which we have addressed (a) the search for efficient visual descriptors, (b) the analysis of the extent to which our heuristic metric resembles the human criteria for relevance and, finally, (c) the experimental validation of our complete proposal. In the experiments, we have used both a public image database and images collected at our research center.

## 1. Introduction

Presently there is an increasing market for service robots which is still dependent on progress to overcome technological limitations. In this environment, the extent to which domestic and personal service robots can recognize scenes will have a direct impact on what these robots can do to assist humans in their daily activities. At the same time, a good balance between cost and performance is crucial. Robots at home aimed at domestic cleaning, home security and surveillance, child and elderly assistance, etc., must not be expensive, which constricts their hardware, sensor, and power capabilities, and consequently the control software embedded within them.

Since collecting geo-tagged datasets is time-consuming and labor-intensive, and indoor places do not necessarily have GPS (Global Positioning System) information, image-based place recognition and localization has been attempted for indoor environments in recent years [1,2]. Taking advantage of the great deal of research and progress made in the field of visual scene recognition [3,4], the basic idea of current image-based place recognition and location approaches in robotics is to search a database of indoor images and return the best match [5,6].

The research described in this article is concerned with supervised place recognition (scene categorization) from static images in robotics. This means extracting visual features from any input image, and then using a trained classifier (e.g., a Support Vector Machine) to identify it as belonging to one particular category. In this kind of task, feature extraction plays a vital role, and is one of the first issues addressed. An overview of both hand-crafted and deep-learning-based descriptors widely applied to place-recognition tasks can be found in [1].

Another important aspect concerning this task is related to the image acquisition rate: most robots have a camera or an RGB-D sensor on board, from which images are taken to identify the scene. Nevertheless, either the robot takes frames at a fixed rate that is often predetermined, or it captures the images whenever for a reason dependent on the task or the environment, i.e., the scene in this case needs to be identified. The problem in both cases lies in the fact that quite often the robot ends up with meaningless or noisy images which reduce the performance of the classifier [7].

In this work we will describe a novel strategy for scene recognition that will determine automatically when an image contains enough information either to train the classifier or to identify the scene. This strategy will help to maintain a high performance of the classifier by avoiding noisy or meaningless images, as simulation studies for several Convolutional Neural Netwok (CNN) architectures have suggested [8]. This may be also interpreted as an automatic way of fixing valid viewpoints or imposing viewing restrictions. Specifically, we make the following contributions:

1. A novel measure of image relevance based on its coverage of an indoor place.
2. An approach to indoor scene categorization able to incorporate information about image relevance/coverage.
3. An experimentation to analyze the correspondence between our metric and human perception of image relevance.
4. An experimentation to determine whether the use of the coverage metric is useful to solve the recognition problems based on both hand-crafted and learned features.

According to all this, and after a brief Related Work subsection, we will first analyze different visual descriptors suitable for scene categorization in mobile robots and, on a second stage, we will describe our novel automatic strategy to filter out irrelevant images and work only with the most meaningful ones. The impact of this strategy in the performance of the classifier will also be analyzed in a section dedicated to showing the experimental results. Finally, we will summarize the main conclusions and future research lines.

### Related Work

Indoor place recognition is very challenging because of several reasons, among them that different indoor places in the same building usually share similar objects and structural elements. To tackle this problem of ambiguity, multi-sensor information fusion is widely used as in [9], where a multi-sensor-based indoor global localization system, integrating a coarse CNN-based place recognition with a probabilistic approach for fine localization, was proposed.

To reduce the dependency of external hardware resources, Structure-from-Motion (SfM) reconstruction techniques have been used to build spatially oriented 3D models to match 2D query images against parts of the 3D reconstruction model [5]. However, the main requirements of indoor place recognition for robotics include high speed in real time. Therefore, alternative approaches collect randomly distributed views in each room from the training images, and then match the information of query images to the collection to detect the common part and recognize it [7].

Though deep models have achieved promising results in place recognition, their requirements for high amounts of images for training have led to some authors looking back to hand-crafted features and eliminating the image-based training phase encountered in traditional place-recognition algorithms [10].

Regardless of the type of learned or hand-crafted features that are used, common objects and structural elements between places cause wrong recognition. To avoid this potential mismatching, is necessary to eliminate similar views belonging to different categories, which are quite often the least representative of the real places [8]. To the best of our knowledge, ours is the first method proposed for automatic selection of the most relevant views for both indoor place scene description and query. The approach presented in this paper can be easily combined with any method of the state of the art as a pre-processing stage.

## 2. Materials and Methods

### 2.1. Visual Descriptors for Scene Recognition

One of the key aspects to achieve a good performance in scene recognition is the selection of good image descriptors, hence we decided to investigate the performance of classifiers when working with different representations. A good visual descriptor must possess discriminant power to characterize different semantic categories, while remaining stable in the presence of inter and intra-class variations caused by photometric and geometric image distortions [3]. There are many visual descriptors which are well known in the field of scene recognition [3,11]; the most common traditional options are either local or global features, with which impressive results have been achieved. Nevertheless, during recent years a new powerful image-representation extractor based on CNN has appeared which offers the best state-of-the-art performance in scene recognition [1,12,13]. CNNs are inspired by the discoveries of Hubel and Wiesel regarding the receptive fields in the mammal visual cortex. The CNNs are based on three key architectural ideas [3]: (1) local receptive fields for extracting local features; (2) weight-sharing for reducing network complexity; (3) sub-sampling for handling local distortions and reducing feature dimensions. Therefore, a CNN is a feed-forward architecture with three main types of layers used to map raw pixels to image categories: (1) 2-D convolution layers which implements adjustable 2-D filters—the output of each filter is called a feature map; (2) sub-sampling or down-sampling layers which reduce the dimensional of each feature map but retains the most important information; and (3) output layers to perform the labeling of the image. Recent studies show that general features extracted by CNNs can be transferable and generalized to other visual-recognition tasks [1,14], in fact, since designing and training a CNN are computation-intensive tasks, one common option to take advantage of the power the new deep-learning techniques is to use a pre-trained CNN without the last fully connected layers, as feature extractor. In [15] the authors propose using visual saliency to enhance relevant information and the deep features (using a pre-trained model) from multiple layers of the CNNs for classifying scene images. The authors in [8] propose a model that combines several CNN architectures with a multi-binary classifier. As opposed to these methods, where CNNs are merely used as feature extractors, in [16] a Naïve Bayes Nearest Neighbor (NBNN) is integrated with a CNN framework in a deep network that is end-to-end trainable.

The experimental results section will describe the performance of a classifier when using the traditional global and local descriptors, as well as the combination of both, and deep-learning-based ones.

Regarding the first option (local descriptors), the most usual approach consists of selecting some salient points in the image (using SURF, SIFT or similar strategies) [11,17], and then building a description from this unstructured set of points using a bag-of-visual-words [18]. This process works on three stages: (i) Detection of the local salient points together with their descriptors—each salient point has an associated feature vector; (ii) determination of the clusters into which the descriptors are projected: the clusters are called *visual words*, therefore the projection of a feature vector corresponding to a salient point into a cluster is equivalent to saying that the image contains a specific word; (iii) description of the image as a collection of words: the image is represented by the histogram of the visual words that it contains.

With respect to global descriptors, they summarize the whole image in a single vector, thus reducing the dependence on image-specific details. The first global descriptor we have considered is the combination of Local Difference Sign Binary Patterns (LSBP) and Local Difference Magnitude Binary Patterns (LMBP) [19]. The LSBP is non-parametric local transform based on the comparison among the intensity value of each pixel of the image with its eight neighboring pixels. However, in the case of the LMBP, this comparison is based on a threshold, i.e., whether the intensity differences between each pixel and all its neighbors are higher than a certain threshold. After this, we also applied *spatial pyramids* [20], which in this case means partitioning the image into increasingly fine sub-regions and then computing the combination of LSBP and LMBP inside each region. The representations obtained for all regions are concatenated to form an overall feature vector. To avoid very high-dimensional descriptors, we used principal component analysis (PCA) to reduce the size of the LSBP+LMBP combinations. Therefore, using LSBP+LMBP+PCA+ SpatialPyramids we end up with a 2480-dimensional feature vector for each image: We will call this representation LDBP (local difference binary pattern).

Another global representation that we have considered is the gist of scene [21], which divides the image into a 4×4 grid, filtering each cell using a bank of Gabor filters, and then averaging the responses of these filters in each cell.

Finally, regarding the deep-learning-based techniques, we have used pre-trained CNNs, without the last fully connected layers as feature extractor. Here we used two different architectures from the scene recognition state of the art: VGG-16 [22] and ResNet152 [23]. The output feature vectors are 4096- and 2048-dimensional, respectively. These two CNNs were trained using a combination of ImageNet [24] and Places-365 [25] datasets, resulting in a total of ≈9.2 million images (1.2 + 8).

### 2.2. Detection of Meaningful Images

As pointed out in the introduction, there is an important remark to be considered when working with scene recognition, and that is related to the dependency of the view used to capture the images. This problem is even worse in the specific case of mobile robots where the use of constant and predefined frame rates might cause the acquisition of meaningless images, not to mention the poor-quality images (blurred, occluded, or images taken under difficult illumination conditions) and which are either consequence of the movement of the robot and/or the use of a constant frame-rate. We know that some observations are more suitable than others to identify the scene [26,27]; in fact studies of large photo databases led Simon et al. to discover that different photographers tend to select the same view when taking photos of the same location, thus suggesting that there is a good agreement on the “best” views of scenes [28], so that recognition performance is better for experienced views than novel views. In particular, Mou and McNamara [29] suggest that people use intrinsic frames of reference to specify locations of objects in memory. More specifically, the spatial reference directions which are established to represent locations of objects are not egocentric but intrinsic to the layout of the objects.

**Figure 1:**
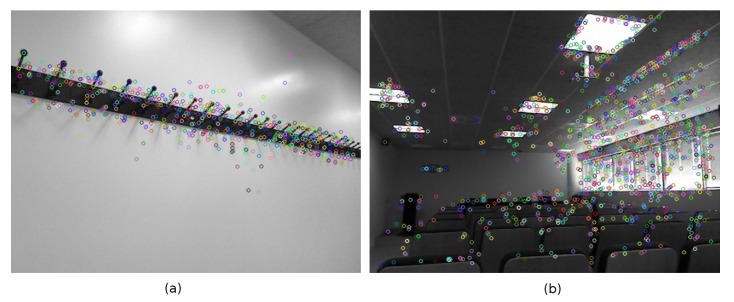
(**a**) Concentrated key points in an image with few objects. (**b**) High scattering of key points in an extensive view.

**Figure 2:**
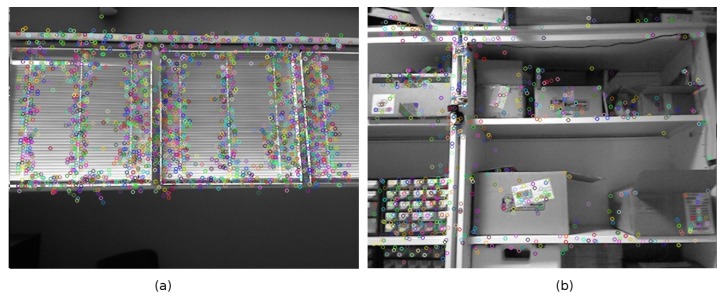
(**a**) A narrow view showing many key points on many close objects. (**b**) Close view but with high scattering of interest points in 3D.

To improve the performance in scene recognition for the particular case of mobile robots, we have opted for the development of a metric able to determine when an image is “rich” enough, i.e., it contains enough information to identify the scene. This metric will be used to filter out meaningless or noisy frames, retaining only the relevant images. This proposal is somehow equivalent to limiting the number of views that are valid to perform the recognition of the scene. Our metric is inspired in the concept of canonical views [27,28,30,31,32], in particular in one of the three characteristics that these images are supposed to fulfill: coverage. Our metric will try to rank the images according to their content of information and particularly based on the distribution of key points across the image. Our metric prioritizes those images that contain relevant key points scattered all over it. If there are few objects the saliency points will be concentrated in a small region (Figure 1a); however, extensive views tend to include more objects and get a high scattering of key points (Figure 1b). Unfortunately, high scattering of saliency points does not necessarily mean far or extensive views; therefore, the metric also must prioritize the presence of far objects (Figure 2). RGB-D sensors provide valuable distance information which complements the one provided by intensity features.

Since we need to deal with distances, we will assume that we will work with the information provided by an RGB-D sensor (such as the Kinect or the Asus Xtion Pro Live). Therefore, in addition to an RGB image we also get a depth map which provides, for every pixel, information about the distance from the sensor. Therefore, not only do we have an image of the scene but also a point cloud in the 3D space. This is the reason we have used a key point detector specifically designed for 3D meshes: Harris 3D [33]. We are aware that other options such as NARF [34], or even different deep-learning architectures for saliency [35,36], can be used. Nevertheless, we assume that more important than the method applied to determine saliency is how these salient points or regions are scattered in the image, which principally helps to determine when the image is representative of the scene.

Considering all this, we designed a heuristic metric to determine the relevance, coverage, or *representativeness* of an image, made up of two terms:

(1) $$\begin{array}{c} coverage\_metric=\sum_{i=1}^{M}\left(H\left(NK\left(Q_{i}\right)-T\right)\right)+\ldots \\ +\frac{1}{M}\sum_{i=1}^{M}\frac{1}{n\_pixels}\sum_{j=1}^{NK(Q_{i})}\left(\frac{d_{j}\left(Q_{i}\right)}{max\_range}\right) \end{array}$$

where *M* is the number of grid regions $Q_{i}$ in which each RGB-D image is divided, $NK(Q_{i})$ is the function which returns the number of Harris 3D key points detected in $Q_{i}$, *T* is a threshold, H(·) is the Heavised step function, $d_{j}\left(Q_{i}\right)$ is the depth of key point *j* in $Q_{i}$, and n_pixels and max_range are normalizing parameters, the number of pixels in each grid region and the maximum grid depth, respectively. The first term of the equation favors images with a big number of scattered key points, while the second term favors images with a high proportion of distant key points. As a whole, this metric favors images with a good coverage of a scene, as explained in detail hereafter.

**Figure 3:**
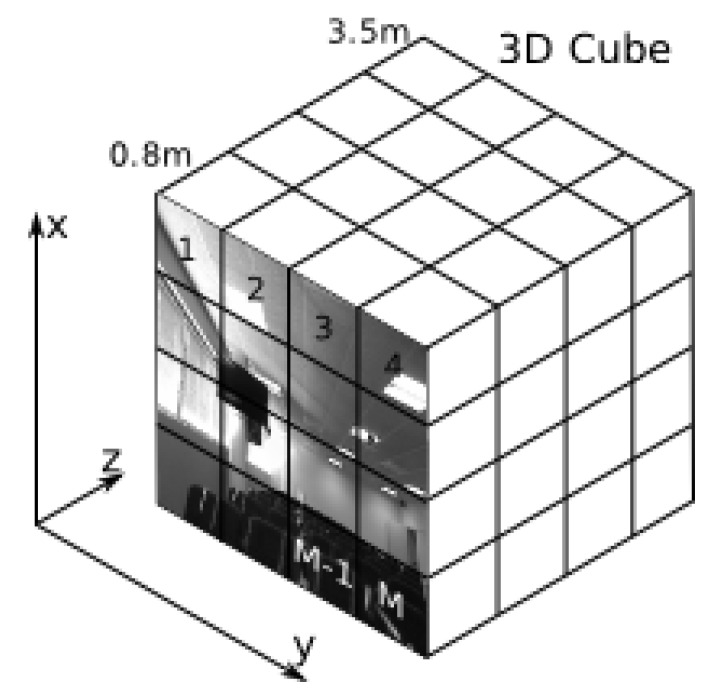
Representation of an RGB-D image as a cube. The XY plane is divided into a set of M-regions.

When Harris 3D or NARF 3D are applied to detect the interest points in the image, the use of our metric involves dealing with the image as a cube instead of a 2D surface (Figure 3), in which the Z-axis represents the distances at which the different points can be detected (in our case from 0.8 m to 3.5 m). The first term of our metric reflects up to what extent the key points are scattered all over the image. Thus, if we divide the XY plane (i.e., the image, Figure 3) into a set of M-regions, the value of this first term, within the interval [0,M], determines how many of these small regions contain more than T key points; $NK(Q_{i})$ represents the number of key points detected in the *i*th quadrant of the XY plane, while $H(NK\left(Q_{i}\right)-T)$ is the Heavised step function:

$$H\left(x\right)=\left\{\begin{array}{cc} 0 & \mathrm{if}x<0\\ 1 & \mathrm{if}x\geq 0 \end{array}\right.$$

The second term of our metric tries to point out those images that contain far objects in the scene. In this second term $d_{j}\left(Q_{i}\right)$ represents the distance to the interest point *j* in quadrant *i*. This second term considers the percentage of pixels in each one of the M-regions of the image that are *distant salient points*, i.e., key points that are detected far from the sensor (in the Z-axis). On the other hand, this second term tries to give priority to those regions which contain a high percentage of key points.

Regarding the parameters of this metric, a grid search procedure will be enough to set the number of regions *M* in which each RGB-D image is divided, and the threshold *T*. It is enough to build a very small validation set in which images are sorted in two groups, relevant or noisy. Next the values of *M* and *T* are searched so that the images with a high score according to the coverage metric (among the 33% best-valued) should tend to be the same as the ones labeled as relevant in the validation set, and on the contrary, the images labeled as noisy should have a low value according to the coverage metric. In our case, the size of the images we work with is 640 × 480 pixels, the value of *M* is 84 (3 × 4 × 7), and the threshold *T* is 3.

### 2.3. Design of a Classifier Able to Incorporate Information about Image Relevance/Coverage

Now we will describe how we can use the coverage metric described in the previous section to limit the number of viewpoints from which we try to recognize the scene. We need a strategy able to set automatically when the camera has just acquired one of these valid views, in the sense that it contains enough information and hence it is possible to proceed with scene recognition.

**Figure 4:**
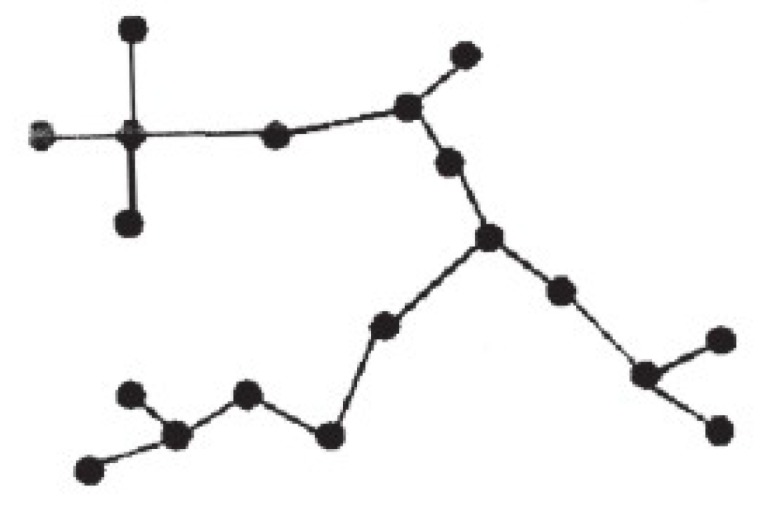
Example of a Minimum Spanning Tree.

First, during the *training stage*, all images included in the *training set* are evaluated according to our metric. Thus, we sort and subdivide the training images belonging to the same class in three categories—Q1, Q2, and Q3—where Q1 contains all images of the training set belonging to the same class and which are among the 33% best-valued, while Q2 and Q3 represent the second and third thirds (and this is done for every class). After this, we compute the Minimum Spanning Tree (MST) [37], which connects all the images (nodes) of the training set, making up a graph without cycles which verifies that the sum of the distances among the connected nodes is the minimum among all possible trees that can be formed from the same set of nodes. The fact that there are no cycles means that there is only one path among any two nodes in the tree (Figure 4).

Every node of the MST stores three pieces of information: the descriptor of the image (a 3080-dimensional descriptor concatenating LDBP and SURF vector features), the coverage value (according to our metric), and finally, whether the represented image belongs to Q1 or not. Nodes are connected by edges while weights are the distances between them. This distance is defined as the absolute value of the dot product of the image descriptors of the pair of edge-connected nodes.

Once a classifier has been trained and the MST created, for any new image we will look for the edge of the MST that is closest to this new image [38]. If the two nodes of the nearest edge are relevant (i.e., within Q1), plus their coverage values are lower than the coverage value of the new image, we conclude that the new image is valid to identify the scene (this only means that this image will be passed through the classifier). This new image will be also accepted for classification when only one of the two nodes of the MST’s closest edge belongs to Q1, but its coverage value is lower than the coverage value of the new image. Finally, if none of the two nodes of the MST’s closest edge belong to Q1, the new image is automatically discarded for labeling. In the experimental results we analyzed the performance of this criterion, but we also considered another alternative which is a bit more flexible; when both nodes connected by the MST’s closest edge are relevant, i.e., belong to Q1, the new image is automatically accepted for its classification.

## 3. Results and Discussion

### 3.1. Analysis of Different Image Descriptors

Given the importance of the image descriptor in scene recognition, our first set of experiments have aimed at the analysis of the performance achieved with a Support Vector Machine, when it works with the different image descriptors mentioned in Section 2. For this purpose, we have created an image set obtained at our research center. We have divided our images in 11 different classes: offices, different laboratories, staircase, common staff areas, assembly hall, kitchen, etc. These images were taken on different days at different hours, and under different illumination conditions: daylight (natural illumination), artificial illumination only, and half-light (penumbra or semi-darkness). The class with the shortest number of images has 170 samples.

Considering the results shown in Table 1, we can easily realize one of the deep-learning-based techniques, VGG ranks first, although ResNet also performs well. In the case of the traditional descriptors, both global and local, it is the combination of both the options which provides the best performance: in our case the joining of a holistic representation (the local difference binary pattern, LDBP) together with the visual bag of words (local representation based on the use of SURF).

The SVM uses an RBF kernel. The software used is the SVM implementation in OpenCV based on LibSVM. To train the SVM with optimal parameters, a grid search is performed to determine the gamma parameter using a grid search and a 10-fold cross-validation. This is the procedure that has been followed to train all the SVMs described in this section of experimental results.

**Figure 5:**
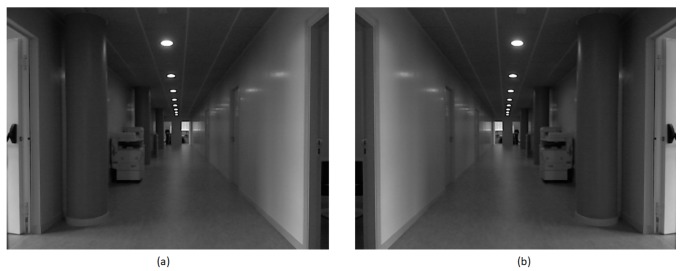
Original test image on the left, and new test image, obtained from the original one by mirror reflection, on the right.

We have also analyzed the robustness of the SVM classifier on recognizing mirror images. In particular, we have doubled the size of the test set, by adding images that were taken from the original ones by mirror reflection. Figure 5 shows how the test set is augmented with mirror reflections of original test images. In this case, the training and validation data sets are still the same as before, and only the test set has been altered by including the extra images obtained from the original ones by mirror reflection. Table 2 shows the experimental results. The results confirm the robustness of the descriptors that best performed before (VGG, ResNet, and the combination of LDBP and visual bag of words with SURF). This kind of analysis is very useful, since a class such as office represents environments which are the same (regarding type of furniture, size, etc.), but it can happen that the arrangement of furniture in one of the offices is symmetrical to the arrangement of the same furniture but in other office. In this case, it would be desirable that both rooms are identified as “offices”.

### 3.2. Analysis of the Correlation Among Our *Coverage Metric* and Relevance from a Human Perspective

In this second experiment, we wanted to analyze to what extent our *coverage* metric (Section 3) represents what the human would consider as relevant. In this case, we use a new dataset with images collected at our research center with an Asus Xtion Pro Live (RGB-D sensor). To simulate real conditions, we collected data using a constant frame-rate acquisition of 0.5 Hz. We kept the cloud points provided by the RGB-D sensor so that we could apply the Harris3D operator and our metric Equation (1). The training set is made up of 515 images, while the test set is formed by 884 images. In this new data set, there are 5 classes (the number of training images per class is shown in the first column of Table 3).

**Figure 6:**
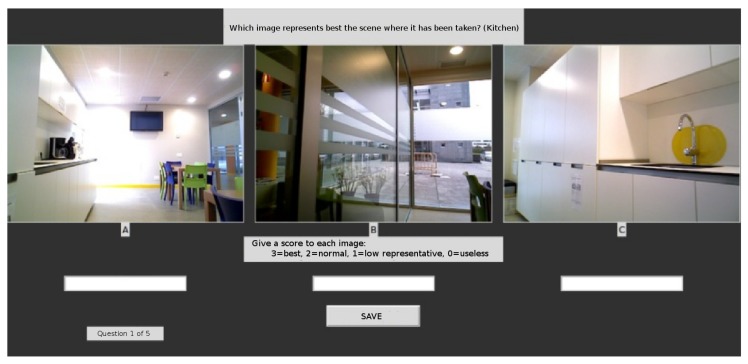
Snapshot of the screen taken during the performance of the second experiment, while taking a poll.

To analyze the correspondence among our metric and people’s opinion we have designed a poll that could be carried out in a short period of time, while trying to reduce the degree of noise usually inherent to these kinds of surveys. First, we sorted the training images for every class using our metric (Equation (1)), next we built the three sets mentioned in Section 4 for every class: the set with the images in the first third (Q1), according to our metric, and the sets with the images in the second (Q2) and third (Q3) thirds. Once this has been done, for every class, different people were presented with three images, and each one of them was randomly picked from each one of the sets Q1, Q2, Q3 (no information was given to the voter about this). As depicted in Figure 6, each user was shown three pictures of the same scene. The user had to give a score to every picture, thus selecting the most representative, the second most representative and so on. The user also had the chance to determine that an image was completely meaningless. The voter had to give a score to each one of these three images. The score could be 3 points (very representative), 2 points (medium degree of representativeness), 1 point (low representativeness), or 0 points (meaningless, the image does not provide information at all). As we can see in Table 3, 30 volunteers participated in the poll. They were young people, mostly 17 and 18 years old, who were not related or familiarized with the environment were the data set was collected. Each one of them carried out the poll individually and isolated from the other participants to avoid biases or mutual influences.

**Figure 7:**
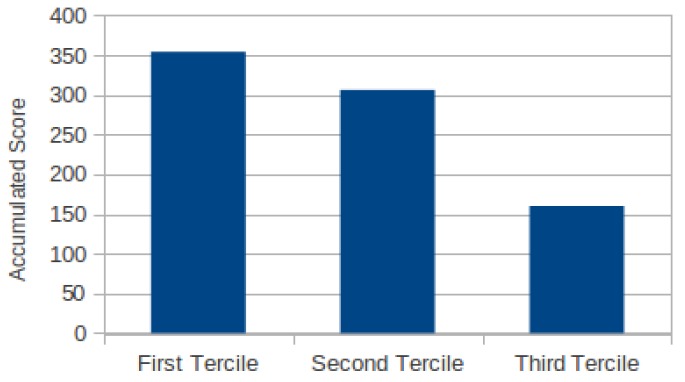
Score accumulated by the images in the first third, second third and third according to the raking obtained using our metric.

Figure 7 summarizes the results of the experiment. As we can see in this figure there is a clear positive correlation among human-relevance and our metric. The images in Q1 (highest relevance according to our metric) got the highest sum of scores, the next highest sum of scores was for Q2, and finally Q3 contains the images less valuable according to our metric and the people who participated in the experiment.

### 3.3. Improvement of the Performance of the Classifier Thanks to the Coverage Metric

In this last experiment, we have tried to determine whether the use of coverage metric is useful to solve the classification problem; i.e., the recognition of the scene. In particular, we analyzed the performance of a classifier when it is trained under different scenarios (Table 4): (a) using all the images in the training set (515 images in total); (b) using only the most important training images according to our metric, Q1, first third of the images sorted by their importance (170 training images); (c) considering the training set formed by the images that were selected as “best representative” by any of the persons who participated in the poll (115 images in total, Q1-people in Table 4). Each one of these three models was tested with the whole test set (884 images), and with the set formed by only the most relevant test images according to our metric (test images in the first third considering their coverage value, 293 images).

As we can see through the results, the performance increases in all cases when only the most relevant images of the test set are being labeled (Q1-images), as shown in Table 5. In fact, the best combination is the one obtained with a model specifically trained with the Q1 training images and then also tested with Q1 testing images. It seems that this model is less robust when it must identify noisy or not so meaningful images, but we must remember that this model has also been trained with a reduced number of training images. Obviously if the model is supposed to label all images it must be trained with noise too. Nevertheless, what seems to be clear is that the best combination seems to be a model specifically trained to recognize scenes under valid viewpoints.

Finally, given the promising results, we decided to apply the strategy described in Section 4, based on the combination of a Minimum Spanning Tree together with an SVM classifier. The MST filters out the irrelevant images, while the SVM performs the recognition of the scene using only meaningful images (Table 6). The first three columns of table (Table 6) show the performance of an SVM classifier trained only with the relevant (labeled) images of the training set (Q1 training images). The first column represents the ideal situation, i.e., the model is used to test those images that we know that are relevant according to our metric (Q1-test images). Nevertheless, this situation is not realistic, i.e., normally we do not have a test set beforehand to choose the best images to be labeled. During a normal operation, the model will get an image and will have to decide whether classify it or not. In this case, we have introduced an MST which is used first to determine whether the image is meaningful enough, and if so, classify it using the SVM. Columns 2 and 3 of Table 6, show the performance achieved under this working scheme. We analyzed the performance when the MST works with the two possible heuristic rules described in Section 4 for determining when an image is relevant enough (the most restrictive option means that a test image is only relevant when its coverage value is higher than the coverage values for the nearest training images of the MST, this restriction about the coverage values does not apply for the relaxed filter). As we can see, the most restrictive set of rules filters too many images. The best option is using the MST with the least demanding set of rules.

As we can see in Table 6, the number of images correctly classified is still high and better than working will all testing images (fourth column of Table 6), or even better than the normal situation, where no MST or metric is used to determine which images are relevant to perform the task of scene recognition (fifth column of Table 6). Nevertheless, in this case the least restrictive heuristic rule exhibits the best behavior since the performance of the SVM is roughly the same in both cases but the MST filters out less images that might be relevant according to our metric. As we can see, using our metric and the MST, our method reduces drastically the number of images erroneous labeled, by automatically discarding images that do not seem to be clear enough. However, it is important to realize that this decision is taken by the combined use of our metric and the MST.

## 4. Conclusions

In this paper, we have addressed the problem of scene recognition, particularly oriented for the case of mobile robots. First we reviewed some of the most common image descriptors, and we analyzed their performance using images collected at our research center. From the results we can conclude that the combination of global (LDBP) and local representations (SURF), or some deep-learning-based descriptors (VGG and ResNet), seem to be the best option. Nevertheless, there is still an important problem that must be tackled, and that is the fact that scene recognition is view-dependent. This problem is particularly bad in the case of a mobile robot due to the use of constant sampling rates, or the fact that each acquisition is carried out without any consideration about illumination conditions, occlusion of the scene, etc. To solve this, we have introduced a novel metric with the purpose of limiting the number of valid viewpoints. Our metric tries to prioritize images that contain salient points scattered all over the image, and which contain both close and far objects. The experimental results show a positive correlation among our metric and what some people consider as relevant or significant. Finally, we have used a Minimum Spanning Tree combined with an SVM to automatize the determining of when an image is representative enough for scene recognition. Through the experimental results we confirmed that the performance of the classifier is increased when either the metric or the MST is used only to identify the subset of relevant images.

## Figures and Tables

**Table 1:** Performance of an SVM Classifier using different feature representations. These results are the average performance after using four test sets (4-fold cross-validation). The 3-top scores are shown in bold.

Representation	All Images
	11 Classes
LSBP	78.75%
LDBP	80.78%
LMBP	59.54%
Gist	77.35%
SURF	77.48%
LMBP+SURF	79.07%
LBP+SURF	**87.71%**
Resnet	**83.33%**
VGG	**88.46%**

**Table 2:** Performance of an SVM Classifier with different feature representations and using augmented test sets with symmetrical images.

Feature	Performance
Representation	Train 11 Classes
Census Transform (LSBP)	72.12%
LDBP(LSBP+LMBP)	73.62%
LMBP	53.80%
LDBP(LSBP+LMBP)+SURF	**81.89%**
Resnet	**86.96%**
VGG	**85.58%**

**Table 3:** Summary of the second data set and participants in the poll.

Class	Images	Voters
[0.5ex]		
0 (Instr. Lab.)	61	30
1 (Laboratories)	92	30
2 Common Staff areas (floor 1 and 2)	144	30
3 Common Staff areas (Ground floor)	122	29
4 (Kitchen)	96	30

**Table 4:** Comparison of the performance achieved with models trained using a either all the images available or a subset of images selected according to their coverage. The performance shown is the one achieved when the SVM classifies **all testing images.**

Training Set	LBP+SURF	Resnet	VGG
all train	88.91%	88.80%	85.52%
Q1-train	79.97%	77.04%	76.92%
Q1-people	82.23%	74.55%	77.15%

**Table 5:** Comparison of the performance achieved with models trained using a either all the images available or a subset of images selected according to their coverage. The performance shown is the one achieved when the SVM classifies the **Q1-testing images.**

Training Set	LBP+SURF	Resnet	VGG
all train	91.80%	94.54%	84.64%
Q1-train	92.15%	92.49%	88.40%
Q1-people	90.78%	84.64%	86.69%

**Table 6:** Performance achieved with the combination MST+SVM for scene recognition described in Section 4. The SVM was trained using only the most relevant training images (Q1). The first column shows the results without the MST (the Q1-test images are used after all test images have been sorted using the coverage metric). The second and third column show the role of the MST automatizing the filtering of images, no sorting of the test set has been carried out in these cases. We can also see how many of the 293 relevant images pass the MST filtering. The percentages are given with respect to the total number of images of the test set that have been labeled by the SVM.

Descriptor	Q1-Test	Restrictive Filter	Relaxed Filter	All Test	All Training and All Test
	train = 170 im.	train = 170 im.	train = 170 im.	train = 170 im.	train = 515 im.
	labeled = 293 im.	labeled = 76 im.	labeled = 205 im.	labeled = 884 im.	labeled = 884 im.
LBP+SURF	right = 270	right = 68	right = 184	right = 707	right = 786
	wrong = 23,(7.8%)	wrong = 8,(10.5%)	wrong = 21,(10%)	wrong = 177,(20.0%)	wrong = 98.0,(11.09%)
Resnet	right = 271	right = 69	right = 173	right = 681	right = 785
	wrong = 22,(7.5%)	wrong = 7,(9.2%)	wrong = 32,(15.6%)	wrong = 203,(23.0%)	wrong = 99,(11.2%)
VGG	right = 259	right = 70	right = 183	right = 680	right = 756
	wrong = 34,(11.6%)	wrong = 6,(7.8%)	wrong = 22,(10.7%)	wrong = 204,(23.1%)	wrong = 128,(14.5%)
